# A Policy Analysis on the Proactive Prevention of Chronic Disease: Learnings from the Initial Implementation of Integrated Measurement for Early Detection (MIDO)

**DOI:** 10.15171/ijhpm.2017.18

**Published:** 2017-02-20

**Authors:** Roberto Tapia-Conyer, Rodrigo Saucedo-Martínez, Ricardo Mújica-Rosales, Héctor Gallardo-Rincón, Evan Lee, Craig Waugh, Lucía Guajardo, Braulio Torres-Beltrán, Úrsula Quijano-González, Mauricio López-Mendez, Elena Rose Atkinson

**Affiliations:** ^1^Fundación Carlos Slim, Mexico City, Mexico.; ^2^Eli Lilly and Company, Lilly Global Health, Geneva, Switzerland.; ^3^Lilly NCD Partnership, Indianapolis, IN, USA.; ^4^Lilly NCD Partnership, Mexico City, Mexico.; ^5^C230 Consultores, Mexico City, Mexico.

**Keywords:** Early Diagnosis, Mass Screening, Policy, Mexico, Chronic Disease, Diabetes Mellitus

## Abstract

Mexico, like many low- and middle-income countries (LMICs), faces an epidemic of chronic non-communicable diseases (NCDs), specifically diabetes, hypertension, obesity, and lipid disorders. Many people with these NCDs may not be aware that they have a disease, pointing to the need for broader screening programs. The traditional prevention policy in Mexico was based on screening with a paper-based risk factor questionnaire. However, this was used to screen patients already seeking healthcare services at facilities, and screening goals were set as a function of the number of questionnaires applied, not number of individuals screened. Due to this, Fundación Carlos Slim developed *Medición Integrada para la Detección Oportuna* (MIDO^TM^), or Integrated Measurement for Early Detection, an NCD screening and proactive prevention policy.

This document is a policy analysis based on early learnings from the initial implementation of MIDO in eight primary healthcare centers in two central Mexican states.

MIDO was found to expand screening programs beyond clinic walls, systematize community screening strategies, emphasize the detection of pre-disease phases, incorporate lifestyle counseling, and propose screening goals based on population targets. In collaboration with the Mexican Ministry of Health, MIDO has successfully screened over 500 000 individuals—about 40% of whom would not have been screened under previous policies. Of these more than 500 000 screened individuals, 13.4% had pre-diabetes (fasting glucose between 100 and 125 mg/dL), and 5.8% had undiagnosed diabetes (defined as fasting glucose above 126 mg/dL or random glucose above 200 mg/dL). However, there is still room for improvement in linking positive results from screening with disease confirmation and with patient incorporation into disease management. The experience of implementing MIDO in Mexico suggests that primary and secondary prevention programs in other parts of the world should consider the need for population-based screening targets, a greater focus on pre-disease stages, and the streamlining of the transition between screening, confirmation of diagnosis, and incorporation of patients into the healthcare system.

## Global Situation

Diabetes affects at least 415 million people worldwide and in 2015 accounted for an estimated 12% of global health expenditures.^1^ Low- and middle-income countries (LMICs), with limited infrastructure for diabetes care, bear the burden of 75% of these cases.^2^

LMICs must, therefore, determine which prevention efforts are the most cost-effective for reverting this epidemic and where to allocate scarce resources to achieve maximum impact.^3^ Although policy recommendations on specific regulatory measures to encourage healthier diets enjoy broad consensus,^1^ prevention and screening programs in a clinical setting are not as well-defined.^4^

## The Mexican Situation

Mexico is no exception to this global epidemic; confirmed cases of diabetes in adults over 20 years of age have been rising steadily from 4.0% in 1993, to 5.8% in 2000,^5^ 7.0% in 2006,^6^ and 9.2% in the most recent nationwide survey in 2012.^7^

Other related chronic non-communicable diseases (NCDs)^ [1]^ have experienced similar increases, with the prevalence of obesity rising by almost 60%, and hypertension by almost 30% between 1993 and 2012.^8^

Moreover, confirmed cases may severely underestimate the epidemic: the true prevalence of diabetes was estimated to be 10.9% in 2000,^9^ and 14.4% in 2006.^10^ This suggests that approximately half of Mexicans with diabetes are unaware of their disease status, and therefore require broader screening programs to reach them.

## Policy Context

Since 1999, Mexico’s Ministry of Health’s NCD screening policy has been using a paper risk factor questionnaire in adults above 20 years of age.^11^ However, this intervention is limited—only passively screening established patients of a primary health center (PHC), and it operates without population-based screening goals or up-to-date robust information systems.^12^

In response to the growing NCD epidemic, in 2010 Fundación Carlos Slim (FCS) developed *Medición Integrada para la Detección Oportuna* (MIDO^TM^), or Integrated Measurement for Early Detection. MIDO is an innovative healthcare policy that incorporates proactive primary and secondary NCD efforts into PHCs^ [2] ^.

MIDO is a core pillar of FCS’s CASALUD^TM^, a comprehensive healthcare model (that includes respective practical tools) for NCD prevention and disease management. The other pillars of CASALUD include patient-centered healthcare, monitoring of the medication supply chain, capacity building, and evidence-based disease management.^8,14^

In 2013, the Mexican government launched the National Strategy for the Prevention and Control of Overweight, Obesity, and Diabetes (hereafter referred to as the “National Strategy”) in order to systematize prevention efforts for these diseases.^15^ MIDO was incorporated into the National Strategy as a reference policy for primary and secondary prevention in PHCs, especially in the practical implementation of screening tools and in the ongoing monitoring of screening progress and control over the epidemic. Due to FCS’ alliance with the Ministry of Health, MIDO currently operates, and complements the Ministry of Health’s NCD screening policy, in 138 PHCs in 27 states (out of Mexico’s 32) as part of the CASALUD Model.

Likewise, FCS developed the NCD Integrated Dashboard to monitor the state of NCDs screened and treated under CASALUD. The Integrated Dashboard is a public, transparent website updated on a daily basis at the different health care levels (PHC, regional, state, and national). The Dashboard is an integral part of the Mexican NCD National Observatory that monitors the entire National Strategy^ [3] ^, and allows authorities to make decisions based on robust, real-time data.

## Description of Integrated Measurement for Early Detection

MIDO is a screening and proactive prevention policy carried out through: all-in-one tools for outreach, screening, and counseling in PHCs and at a community level, and a robust information system to facilitate public health planning and patient follow-up. Importantly, MIDO is based on a proprietary and comprehensive framework of the patient engagement continuum, which is adopted by PHCs currently operating CASALUD.

The patient engagement continuum is defined as a three-step process that (1) provides patients with information about their health status (through screening), (2) confirms their diagnosis, and (3) incorporates them into PHCs. It is important to mention that MIDO is designed to operate exclusively within the primary care level, and does not contemplate patients with complications, who should be appropriately referred to secondary or tertiary care centers.

MIDO’s entry point to this patient engagement continuum takes place through 2 different screening tools: the MIDO Mobile Cart for screening at PHCs, and the MIDO Backpack for screening at the community/household level. PHCs are fully autonomous in the use of these tools; they are responsible for establishing the schedule and personnel responsible for their operation in order to fulfill the population screening targets. Both screening tools include equipment to measure waist circumference, height, and weight, a body mass index calculator, blood pressure monitor, and a glucose testing kit^ [4] ^. All measurements are performed on all patients, with the exception of the glucose test, which is performed based on the results obtained from a risk factor questionnaire.

MIDO’s screening questionnaire incorporates the algorithm defined by CENAPRECE, Mexico’s National Center of Preventive Programs and Disease Control, in order to determine diabetes risk to make decisions on glucose blood test screening. MIDO’s questionnaire also incorporates the most recent international evidence-based cardiovascular risk evaluation tools, and is validated in the Mexican context^ [5] ^.

Importantly, MIDO incorporates pre-disease stages instead of merely dichotomizing screened individuals into “healthy” and “sick” categories. Specifically, pre-obesity is defined as body mass index between 25-29.9, pre-hypertension as systolic blood pressure between 130-139 mm Hg or diastolic pressure between 85-89 mm Hg, and pre-diabetes as fasting glucose between 100-125 mg/dL. Healthy individuals are those with values under these ranges, and those with a disease are defined as having values above these ranges. For diabetes, a probable case is defined as fasting glucose above 126 mg/dL or random glucose above 200 mg/dL. After obtaining the results, screened individuals receive personalized health promotion in the form of counseling and pamphlets that include step-by-step instructions on lifestyle changes and time frames for repeat testing.

Both tools incorporate proprietary software, SI-MIDO^TM^, either on a computer for the Mobile Cart, or as an app for a tablet for the MIDO Backpack. Healthcare professionals enter the information of screened individuals into SI-MIDO, which facilitates follow-up by providing a database of the screened population and their results.

Additionally, in order to shift from paper-based records to nominal information systems, FCS worked with the Ministry of Health within the framework of the National Strategy to design, develop, and scale the *Sistema Nominal de Información en Crónicas* (SIC^TM^) or NCD Information System. FCS is currently consolidating the SI-MIDO and SIC information systems, so that health providers and decision-makers can identify those newly screened patients who were then incorporated into, and treated at a PHC.

## Policy Questions and Data Collection for the Analysis of Integrated Measurement for Early Detection

This policy analysis is based on early learnings from the initial implementation of MIDO. The analysis is structured to answer 2 questions: Is MIDO offering solutions to improve screening efforts in PHCs? What policy lessons can be learned from MIDO’s initial implementation? The study was carried out in eight PHCs in 2 central Mexican states, using four instruments specifically designed to answer the 2 questions above mentioned:

- Interviews with high level public health authorities of 2 states and directors of eight PHCs (68 interviews over the course of 2 years).
- Shadowing and interviews of 60 individuals screened with the MIDO Mobile Cart.
- Analysis of 19 community screening events with the MIDO Backpack.
- Analysis of the SI-MIDO database from the two studied states (over 35 000 screened individuals).

Additionally, the researchers obtained information from the Dashboard on screenings carried out nationwide (over 500 000 individuals screened in the 27 states where MIDO operates).

## Findings of Integrated Measurement for Early Detection as a Screening and Proactive Prevention Policy

Our findings from the policy analysis are organized along each step of the patient engagement continuum: population screening, confirmation of disease status, and incorporation of confirmed cases into PHCs. Findings are summarized in Table and then described in detail in this section.

**Table:** The Patient Engagement Continuum: Screening, Confirmation, and Incorporation

**Step of the Continuum**	**Type of Prevention**	**Policy Issue**	**Policy Solution Offered Through MIDO**	**Specific Actions Offered Through MIDO**	**Findings of Policy Analysis**
Screening	Primary and secondary	Screening in PHCs restricted to current patients	Screen all individuals at PHCs, not just established patients	Screening with a MIDO Mobile Cart in PHCs including all individuals present at a PHC.	40% of those screened with MIDO would not have been screened without this policy.
		Minimal community screening	Expand and systematize community screening strategies	Community screening with MIDO Backpack through home visits and at community spaces.	Community screenings reach a more balanced ratio of men to women (33/67) compared to PHC screening (25/75).
		Emphasis on disease	Emphasize detecting pre-disease stages, and prevention counseling	Identification of individuals at risk for developing NCDs (at pre-disease stages, conditions more amenable to prevention and lifestyle changes) and provision of personalized action plans and counseling.	MIDO places emphasis on pre-disease stages through personalized pamphlets and counseling. MIDO screening identified that 13.4% of screened individuals had pre-diabetes (fasting glucose between 100 and 125 mg/dL), and 5.8% had undiagnosed diabetes (defined as glycated hemoglobin levels above 7%).
		Screening goals based on number of performed screening tests	Set screening goals based on number of screened individuals and population targets	Establishment of screening goals as the percentage of screened individuals of the target population in a certain geographical area.	Each PHC has a clearly defined population of responsibility (population-based goal), and progress is measured in a public and transparent manner through the Dashboard. As of December 5, 2016, 548 355 people have been screened, 77.3% of the total goal for 2016.
Confirmation of diagnosis	Secondary	Complicated and lengthy process to confirm diagnosis	Design a lean process for confirmation of diagnosis (towards the design of a one-stop shop)	MIDO proposes that screening, immediate referral to confirmation, and appointment scheduling happen at the same service touchpoint.	(Implementation in progress. Policy analysis limited to conceptual design of the tools.) Preliminary findings suggest that a very low proportion of patients schedule a confirmation appointment after their screening.
Incorporation into PHC and treatment	Precursor to tertiary	Screening efforts not linked to incorporation of patients into the healthcare system	Design a lean process for scheduling first appointment (towards the design of a one-stop shop)	SI-MIDO and SIC will consolidate screening information so decision-makers can track newly screened patients (using SI-MIDO) and assure their incorporation into a PHC (SIC).	(Implementation in progress. Policy analysis limited to conceptual design of the tools.) PHCs do not currently keep track of screened patients and cannot quantify losses between each step of the continuum.

Abbreviations: MIDO‏, *Medición Integrada para la Detección Oportuna*‏; NCD‏, non-communicable disease‏; PHC‏, primary health center.‏

MIDO was found to yield positive results on the first phase of the continuum; specifically, MIDO expands screening programs beyond clinic walls, systematizes community screening strategies, emphasizes the detection of pre-disease phases, and establishes screening goals based on population targets. However, MIDO has yet to show results in the second and third phases of the patient engagement continuum: confirmation and incorporation.

### Step of the Continuum: Screening

#### Policy issue addressed: Screening in Primary Health Centers Restricted to Current Patients

The previous Mexican screening strategy was engaged in prevention only passively, as it carried out screenings exclusively for patients already actively seeking care at PHCs. MIDO changes screening policies by going beyond the screening of symptomatic patients to include the screening of apparently healthy individuals.

This distinction is not insignificant; of individuals screened with MIDO Mobile Cart, 17% were accompanying a patient and 23% were handling administrative or paperwork issues at the PHC. In other words, 40% of those screened would not have been screened in the absence of MIDO.

MIDO broadens the scope of the population screened: as of December 5, 2016, a total of 548 355 individuals had been screened at the 138 PHCs that use MIDO, as per the Dashboard.^16^ Of this screened population, 13.4% of the individuals had pre-diabetes (fasting glucose between 100 and 125 mg/dL), and 5.8% had undiagnosed diabetes (defined as glycated hemoglobin levels above 7%). These numbers show the importance of engaging the PHC health system in a discussion on the benefits of population-based screening strategies.

#### Policy Issue Addressed: Minimal Community Screening

In previous years, screening activities were primarily based out of PHCs. MIDO has shown that screening strategies can reach a larger population once they move beyond the walls of health care clinics, and that community screening and broad outreach strategies should be standardized, systematized, and incorporated into the public healthcare system.

The MIDO Backpack allows PHCs to perform home visits and/or screening campaigns in community spaces, including community centers, schools, and private and public institutions. Five of the eight observed PHCs performed household visits or convened public events, in 2014 and 2015, leading to 1980 individual screenings. PHCs that performed screenings in the community and households had a more balanced ratio between women and men (67/33) than PHCs that only performed screening at PHCs (75/25). Screening in community spaces reduces barriers to accessing preventive screening services, and increases access for people (mainly men) who otherwise would not have interacted with the healthcare system.

#### Policy Issue Addressed: Emphasis on Disease

Prior to the National Strategy, Mexico’s screening policy focused on detecting existing diseases, thereby neglecting detection of pre-disease stages. In contrast, MIDO emphasizes the importance of screening for pre-disease stages and providing feedback (pamphlets with personalized action plans and lifestyle counseling) to individuals with pre-disease conditions. Because of MIDO, 13.4% of the screened population now knows that they have pre-diabetes (fasting glucose between 100 and 125 mg/dL). This segment of the screened population received a call to action through appropriate health promotion and lifestyle counseling to proactively prevent NCDs before they develop.

#### Policy Issue Addressed: Screening Goals Based on Number of Performed Screening Tests

The goal of the National Strategy and of MIDO is to screen 100% of people above 20 years of age within the geographic boundaries of each PHC, therefore marking a shift away from traditional screening goals based on the number of tests performed. Screening metrics calculated with the proportion of individuals screened are essential, as under the traditional strategy, multiple tests could be performed on the same person. These screening targets based on the total adult population of specific geographical areas under the jurisdiction of a corresponding PHC promote PHC ownership over the screening activities. Each PHC is responsible for carrying out the appropriate screening activities to fulfill their population goal, which is publicly available in the Dashboard.

As of December 5, 2016, the Dashboard shows that 548 355 people have been screened, representing 77.3% of the general goal for screening 709 811 individuals by the end of the 2016.^16^

### Steps of the Continuum: Confirmation and Incorporation

#### Policy Issue Yet to Be Improved: Complicated and Lengthy Process to Confirm Diagnosis

Despite the progress that MIDO has made in improving the first step of the patient engagement continuum, many PHCs continue to struggle with operational and bureaucratic barriers. These factors often prevent patients from reaching the next step in the engagement continuum: disease confirmation.

Once a screened individual is told they have a probable NCD, they must navigate complicated and time-consuming appointment scheduling and laboratory testing processes. From the sample of 60 shadowed MIDO users, none of the 25 who were screened and detected with a probable NCD attempted (that day) to schedule a visit. Sixteen of these patients reported that it was because the process was too complicated and/or time-consuming, which is in line with the general opinion of health personnel.

These patients’ negative perceptions are understandable, as under current practices, after an initial screening it takes at least 2 visits to the physician’s office and one intermediate visit to an external laboratory to confirm an NCD diagnosis. MIDO supports the efforts of the National Strategy to create lean processes to convert the steps of the patient engagement continuum into a one-stop shop. Screening, immediate referral to a confirmation lab test, and the scheduling of an appointment with a physician should happen at the same point of service, as shown in Figure.

**Figure:**
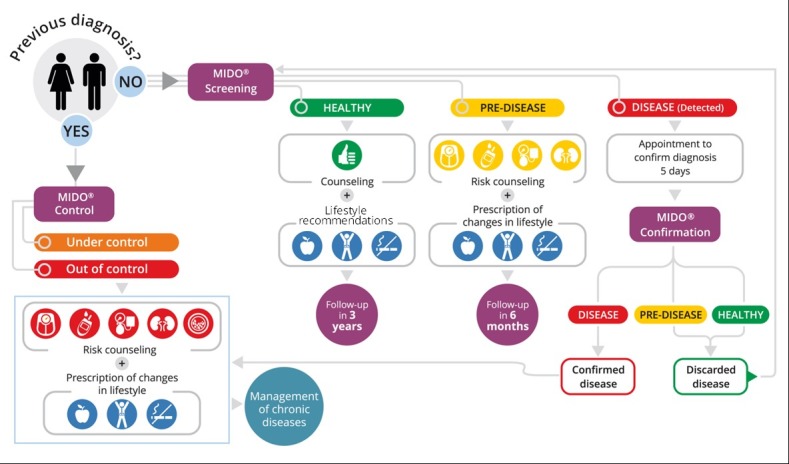
Screening Process With MIDO. Note: MIDO Control is also shown in this diagram. It is an auxiliary tertiary prevention strategy for patients with a previously diagnosed NCD to monitor their condition and screen for other NCDs. Abbreviations: MIDO, Medición Integrada para la Detección Oportuna; NCD, noncommunicable disease

#### Policy Issue Yet to Be improved: Screening Efforts not Linked to Incorporation of Patients Into the Healthcare System

Once patients with a disease or pre-disease have their condition confirmed, they must attend their first medical or counseling appointment as a pre-requisite for disease management. However, PHCs do not currently keep records of newly-detected individuals, nor do they follow protocols to guarantee that these individuals make an appointment with a physician. As a result, the current proportion of patients that are lost-to-follow-up along the continuum is unknown and PHCs cannot currently identify which screened individuals obtain appointments.

FCS is currently consolidating SI-MIDO and SIC to quantify losses between screening and treatment in order to analyze and strengthen the linkages between these activities. By using a unique universal identification number assigned to each person screened and incorporated into a PHC, decision-makers will be able to track how many screened individuals are then treated.

## Reflections on Improving Non-communicable Disease Proactive Prevention Policies

The experience of MIDO suggests that other screening policies worldwide should not neglect three aspects of primary and secondary prevention:

- Screening programs should be measured with population-based targets in order to ensure full coverage and reach a high proportion of the population living with an undiagnosed disease (who may not have medical coverage). All members of a defined geographic area should be screened at least once, with subsequent follow-up screenings according to risk levels.
- A screening policy should contemplate the importance of confirming diagnoses and incorporating patients with a disease into health clinics. Primary and secondary prevention should be seen as a patient engagement continuum that links to tertiary prevention, and efforts should be invested in smoothing the transition and reducing losses between each step.

It is important to not lose sight of what a screening policy should accomplish: the prevention of disease and related complications. Therefore, it is essential that screening policies not only focus on disease, but also on pre-disease stages. Lifestyle counseling for individuals with pre-disease stages is especially important, as these pre-disease stages are more amenable to lifestyle changes.

As may be the case in other LMICs, the heterogeneous nature of PHCs and lack of standardized processes affect the entire patient engagement continuum. For example, the implementation of MIDO suffers from high rotation turnover rates in those responsible for its operation. Of 8 studied PHCs, 7 had appointed a nursing student intern to operate the MIDO Mobile Cart and 1 had appointed a medical school intern. While some student interns were supervised or mentored by a senior nurse, others were not. This lack of standardization is reflected in the quality of screening: MIDO operators only explained results and provided feedback to four of the 25 individuals with a probable disease.

MIDO has strengths as a screening and proactive prevention policy to bridge the gap between the National Strategy and actionable prevention efforts in a clinical setting. However, there is still room for improvement in linking early detection with disease confirmation and patient incorporation into disease management.

The lessons learned from the initial implementation of this policy are valuable for any screening and prevention policy, especially one carried out in a LMIC.

## Acknowledgements

At the national level, Dr. Pablo Kuri, Deputy Secretary for Prevention and Health Promotion at the Ministry of Health, Dr. Jesús Felipe González-Roldán, General Director at CENAPRECE, and Dr. Gabriela Ortíz, Director at CENAPRECE, were key allies in supporting the implementation and evaluation of CASALUD.

Although we cannot list everyone by name here, we appreciate the support shown by the Mexican Public Health System staff along the way. At the state level, Jurisdiction Program Directors Dr. Ma. de la Paz Herrera and Dr. Violeta Nidia Acosta proved invaluable in coordinating with clinics and local authorities. Implementation of the CASALUD Model at healthcare clinics was made possible with the support of their directors: Dr. Carlos Castellanos, Dr. Miguel Ramírez, Dr. Alma Martell, Dr. María González, Dr. Tania Alomia del Río, Dr. Juana Martínez, Dr. Juan Saldaña, Dr. Georgina Sánchez, and Dr. Gloria Vázquez.

We are indebted to the patients and healthcare workers who so graciously participated in tracing studies and interviews at their healthcare clinics. Finally, many thanks go out to our field research team: Christian García, Rayénari Gurrola, Ana Laura Islas, América Ruiz, and Víctor Nolasco, whose tireless data collection efforts made this study possible.

## Ethical issues

Not applicable.

## Competing interests

MIDO is a core component of the CASALUD Model, which was designed, developed and is operated by Fundación Carlos Slim. The analysis reported in this publication was developed in collaboration with and supported by the NCD Partnership of Eli Lilly. The authors Roberto Tapia-Conyer, Ricardo Mujica-Rosales, Héctor Gallardo-Rincón and Rodrigo Saucedo-Martínez are employed by Fundación Carlos Slim in Mexico. The authors Evan Lee, Craig Waugh and Lucía Guajardo are employed full time by Eli Lilly and Company and/or the Eli Lilly NCD Partnership. Braulio Torres-Beltran, Úrsula Quijano-González, Mauricio López-Mendez and Elena Atkinson are employed by C230 Consultores.

## Authors’ contributions

RTC and RMR designed the CASALUD Model and its innovations, conceived the study and helped to draft the manuscript. HGR designed the CASALUD Model and its innovations, conceived the study, participated in its design and coordination and helped to draft the manuscript. RSM designed the CASALUD Model, its innovations, conceived the study, participated in its design and coordination, helped with the framework analysis and drafted the manuscript. EL, CW, and LG helped to draft the manuscript. BTB and UQG conceived the study, participated in its design and coordination, and helped with data analysis. MLM and ERA executed the study, performed data analysis, and drafted the manuscript. All authors read and approved the final manuscript.

## Authors’ affiliations

^1^Fundación Carlos Slim, Mexico City, Mexico. ^2^Eli Lilly and Company, Lilly Global Health, Geneva, Switzerland. ^3^Lilly NCD Partnership, Indianapolis, IN, USA. ^4^Lilly NCD Partnership, Mexico City, Mexico. ^5^C230 Consultores, Mexico City, Mexico.

## Endnotes


[1] MIDO focuses on specific chronic NCDs: diabetes, hypertension, obesity, chronic kidney disease, and lipid disorders. For the sake of simplicity, during this paper, “NCDs” will refer to these diseases listed here.

[2] In this document, “primary prevention” is used to refer to efforts to prevent the occurrence of a disease, “secondary prevention” to refer to efforts to detect or diagnose a disease before signs or symptoms are evident, and “tertiary prevention” to refer to efforts to reduce the impact of a diagnosed disease.^13^

[3] Publicly available at: http://oment.uanl.mx/tablero-de-control-deenfermedades/.

[4] Lipid profiles can also be performed according to each PHCs’ needs and capacity.

[5] The questionnaire includes questions on previous personal and family history of chronic NCDs as well as risk factors, such as smoking.

